# Transoral Approach to Parotid Tumors: A Review of the Literature

**DOI:** 10.3390/curroncol29120740

**Published:** 2022-12-01

**Authors:** Giuseppe Riva, Andrea Lorenzi, Andrea Borello, Andrea Albera, Andrea Canale, Giancarlo Pecorari

**Affiliations:** Division of Otorhinolaryngology, Department of Surgical Sciences, University of Turin, 10126 Turin, Italy

**Keywords:** salivary gland, salivary tumor, transoral approach, parapharyngeal space, parapharyngeal space tumor

## Abstract

Different surgical techniques have been proposed for parapharyngeal space tumors, including transcervical, transparotid, trans-mandibular, infratemporal, and transoral. The choice of the correct approach depends on the size, localization and nature of the tumor. The transoral approach can be used for benign prestyloid masses, such as tumors of the deep lobe of the parotid gland. It guarantees a short hospitalization without skin scars. The narrowed access represents the main limitation of this technique. This review will summarize and analyze the current knowledge about the transoral approach to parotid lesions. Thirty-seven studies were included in a qualitative and quantitative synthesis. The novelty of this review is the quantitative analyses of the clinical data reported in the included studies.

## 1. Introduction

**Figure 1:**
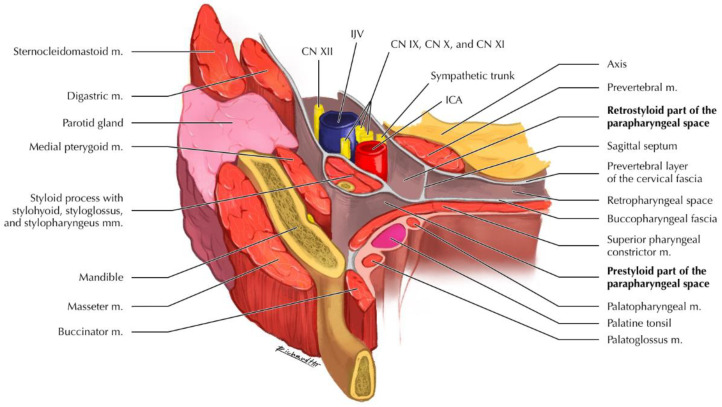
Anatomical representation of the parapharyngeal space. Abbreviations: CN, cranial nerve; ICA, internal carotid artery; IJV, internal jugular vein; m./mm., muscle(s).

Parapharyngeal space (PPS) is located laterally to the pharynx and has the shape of an inverted pyramid extended from the cranial base to the greater cornu of the hyoid bone [1,2]. PPS is divided into prestyloid and retrostyloid compartments by stylohyoid ligament and muscles. Prestyloid space contains the deep lobe of the parotid gland, lymph nodes, ascending pharyngeal artery, ascending palatine artery, and fat, while retrostyloid space contains internal jugular vein, internal carotid artery, cranial nerves (from IX to XII), and lymph nodes (Figure 1) [2].

PPS tumors are uncommon, representing only 0.5% of head and neck neoplasms. Most of them are benign and originate from the deep lobe of the parotid gland [3,4,5]. Magnetic resonance imaging (MRI) with gadolinium and fine needle aspiration cytology (FNAC) represent the gold standard for determining the diagnosis and thus the correct management of the patient. Other radiological examinations may include computed tomography (CT) with contrast, ultrasound scan and magnetic resonance angiography, which is recommended especially for vascularized tumors [6].

Different approaches to PPS tumors have been proposed, such as transcervical, transparotid, transmandibular, infratemporal, and transoral [7,8]. The choice of the surgical approach is based on the type and localization of the tumor and its relationships with neurovascular structures [7,9]. The transoral approach can be used for benign prestyloid masses, such as tumors of the deep lobe of the parotid gland [8].

The transoral approach is performed through an incision near the anterior tonsillar pillar mucosa, thus exposing prestyloid space under the superior pharyngeal constrictor muscle. Stylopharyngeal and styloglossus muscles are important landmarks for the transoral approach to PPS, since they divide pre- and retrostyloid spaces. Therefore, they represent the structures that should not be crossed to avoid severe vascular and nervous damages [10].

This review aims to summarize and analyze the current knowledge about the transoral approach to parotid lesions, with particular attention to tumor features and potential complications.

## 2. Materials and Methods

A review of the English literature was performed through several databases (PubMed, Scopus, accessed on 30 October 2022) to identify articles published before 30 October 2022. A primary search was performed using the terms “parotid AND transoral AND (tumor OR cancer OR adenoma OR warthin)”. Search strategies were adapted for each database. The references of selected publications were then examined to identify further reports that were not found by database searching, and the same selection criteria were applied.

The inclusion criteria were clinical trials, cohort studies, case-control studies, case series, and case reports, regarding the transoral approach for parotid gland tumors. Exclusion criteria were as follows: non-human studies, non-English literature, transoral approach for non-salivary tumors, and insufficient clinical data (i.e., tumor origin) reported in the paper.

The abstracts of all relevant articles were examined using the inclusion criteria for applicability. The references of the selected publications were reviewed to identify further reports that were not found by database searching. Two independent reviewers (AL, AB), working separately, extracted the data from all the eligible studies, which were subsequently cross-checked. All retrieved full-texts articles were included in the review by a consensus of all the authors.

Tumor volume (*V*) was calculated assuming ellipsoid shape (*l* = length, *w* = width, *h* = height) [11]:

$$V=\frac{4}{3}\times \pi \times \frac{l}{2}\times \frac{w}{2}\times \frac{h}{2}$$

If the third dimension (*h*) was not reported, its approximation was [11]:

$$h=\frac{2}{3}\times l$$

## 3. Review of the Literature

**Figure 2:**
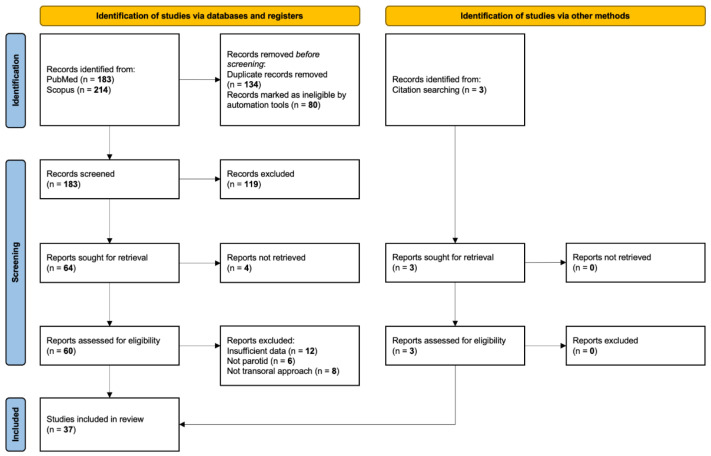
Review of the English literature through PubMed and Scopus, accessed on 30 October 2022. Primary search was performed using the terms “parotid AND transoral AND (tumor OR cancer OR adenoma OR Warthin)”.

A total of 400 published papers were identified through database searches. After abstract screening for eligibility, 63 articles were considered eligible. Among these, we included 37 articles in qualitative and quantitative synthesis after full-text assessment. The other 26 papers were excluded for the following reasons: non-parotid tumors (n = 6), not transoral approach (n = 8), and incomplete clinical data (n = 12) (Figure 2).

Among the papers that matched the inclusion criteria, 14 publications were case reports and 23 were retrospective studies. One hundred and thirty-nine cases were included in the review (Table 1).

Based on available data, the mean age was 49.44 ± 15.73 years (range 14–78 years), with a male/female ratio of 67/72 (48.2% male, 51.8% female). The maximum tumor size ranged from 10 to 90 mm (mean 48.01 ± 15.29 mm, calculated on 116 patients). The maximum size was greater than 50 mm in 59 cases (42.4%). The mean tumor volume, calculated on 100 patients, was 36.52 ± 32.20 cm^3^ (range 0.35–141.99 cm^3^). Therefore, the transoral approach may be a feasible technique also for large tumors of the deep parotid lobe that involve PPS.

**Figure 3:**
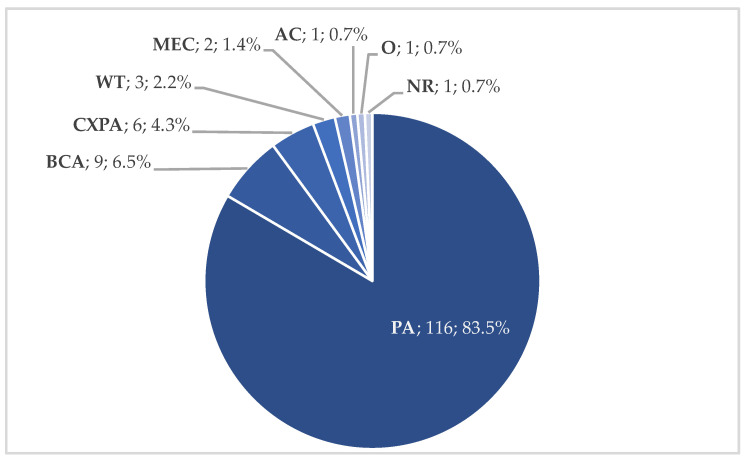
Tumor histology in transoral approach (*n*, %). Abbreviations: AC, adenocarcinoma; BCA, basal cell adenoma; CXPA, carcinoma ex pleomorphic adenoma; MEC, mucoepidermoid carcinoma; NR, not reported; O, oncocytoma; PA, pleomorphic adenoma; WT, Warthin tumor.

Pleomorphic adenoma was reported in most cases (116 patients, 84.1%). Other histotypes were basal cell adenoma (9 patients, 6.5%), carcinoma ex pleomorphic adenoma (6 patients, 4.3%), Warthin tumor (3 patients, 2.2%), mucoepidermoid carcinoma (2 patients, 1.4%), oncocytoma (1 patient, 0.7%), and adenocarcinoma (1 patient, 0.7%). Histology was not reported in one case (0.7%). The transoral approach was used for benign masses in most cases (129 patients, 92.8%, Figure 3). Indeed, benign capsulated masses may be removed through a blunt dissection with a lower risk of injury of vascular PPS structures than malignant lesions that infiltrate surrounding tissues. Preoperative imaging (MRI) is mandatory to evaluate the presence of a tumor capsule or pseudocapsule and correctly select patients for the transoral approach (well-circumscribed masses with a clear cleavage plane from great vessels). Extensive tumors without smooth borders and/or surrounding the facial nerve or major vascular structures may not be suitable for this approach. Moreover, the transoral approach has not been used for retrostyloid masses in the selected studies.

A pure transoral approach was performed in 125 cases (89.9%), while a combined transcervical/transparotid and transoral approach was used in 14 patients (10.1%). Robotic surgery was reported in 55 cases (39.5%) of pure transoral approach and 11 cases (7.9%) of combined approaches. An endoscopic-assisted transoral approach was described in 41 cases (29.5%). Robotic systems and endoscopes were used to improve the visualization of the mass and PPS structures. Indeed, the main limitations of the transoral approach include a poor visualization of PPS major neurovascular structures with possible uncontrollable bleeding, capsule disruption and tumor spillage, and incomplete tumor removal. According to the size and localization of the mass, 0-, 30-, and 45-degree endoscopes were used for the dissection of tumors’ lateral, superior, and deep margins. The development of endoscopy and surgical robotics reduced such disadvantages and increased the feasibility of the transoral approach. However, surgeons must be able to convert the procedure to a transcervical/transparotid approach if needed (impossibility of complete transoral removal, uncontrollable bleeding). Furthermore, trismus is a contraindication for the transoral approach.

**Figure 4:**
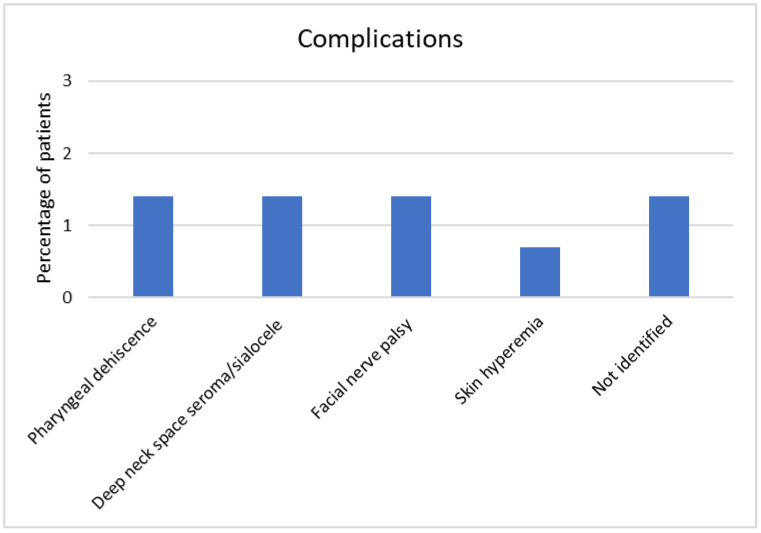
Complications in transoral approach.

Complications were reported in nine cases (6.5%). They were represented by pharyngeal dehiscence (two cases), deep neck space seroma/sialocele (two cases), facial nerve palsy (two cases), and hyperemia of the skin near angulus mandibulae (one case). Two cases described by Boyce et al. had a complication. However, since other patients with non-parotid tumor histology were reported without matching complications, we could not identify those related to parotid tumors (Figure 4) [31]. All the reported complications were solved. No severe bleeding was reported. However, surgeons must be aware of possible severe bleeding from great vascular structures and ready for a prompt transcervical ligation of external carotid artery. A medial displacement of the internal carotid artery should be considered a contraindication for the transoral approach. Severe postoperative pain was not reported in any cases. Wound closure was usually performed in more than one layer to reduce the risk of postoperative wound dehiscence.

The mean follow-up was 26.57 ± 36.58 months (range 1–192 months). However, follow-up was not reported in 31 cases (22.3%). One case of pleomorphic adenoma recurred after 39 months. The low recurrence rate suggests that the transoral approach for selected benign masses has a low risk of capsule disruption and/or incomplete tumor removal. One case of mucoepidermoid carcinoma with positive margins after transoral removal underwent a superficial parotidectomy with traditional open approach. An indication to a pure transoral approach for malignant tumors of the deep parotid lobe involving PPS must be accurately evaluated and reserved to highly selected cases.

The success of the transoral approach for tumors of the deep parotid lobe depends on the correct identification and exposure of the mass to allow the complete removal, prevent recurrence and ensure good functional outcomes, taking into account the risks of PPS surgery.

The main limitations of the published studies include the small number of patients, the short follow-up and the lack of a randomized control group that underwent an external approach.

## 4. Conclusions and Future Perspectives

Selecting the best approach for PPS tumors originating from the parotid deep lobe is based on the volume and extension of the mass to be removed and its putative nature. With these two parameters being established, the less invasive approach that provided sufficient exposure with a low and acceptable complication rate was then chosen. The transoral approach can be selected for benign lesions of the deep parotid lobe that involve prestyloid space. The literature reported a low complication rate with a good recovery in such cases. The novelty of this review was the quantitative analyses of the clinical data reported in the included studies. Further studies with larger samples are mandatory to better understand the role of the transoral approach for PPS tumors with a particular focus on robotic and endoscopic-assisted surgery.

## Figures and Tables

**Table 1:** Transoral approach to parotid tumors: review of the literature.

Reference	Year of Publication	Age (y)	Sex	Length (mm)	Width (mm)	Height (mm)	Volume (cm^3^)	Histology	Approach	Complications/Recurrences	Follow-Up (Months)
Goodwin et al. [12]	1988	66	F	NR	NR	NR	NA	PA	TO	None	120
Goodwin et al. [12]	1988	73	F	NR	NR	NR	NA	PA	TO	None	180
Goodwin et al. [12]	1988	68	M	NR	NR	NR	NA	PA	TO	Recurred after 39 months	39
Goodwin et al. [12]	1988	64	M	NR	NR	NR	NA	PA	TO	None	74
Goodwin et al. [12]	1988	69	F	NR	NR	NR	NA	BCA	TO	None	21
Goodwin et al. [12]	1988	58	F	NR	NR	NR	NA	PA	TO	None	22
Luna–Ortiz et al. [13]	2005	37	M	90	NR	NR	NA	PA	TC + TO	None	19
Rahbar et al. [14]	2006	14	M	NR	NR	NR	NA	MEC	TO	Positive margins, addressed by superficial parotidectomy	13
Bozza et al. [15]	2009	23	M	30	NR	NR	NA	PA	TO	None	192
Bozza et al. [15]	2009	53	F	30	NR	NR	NA	PA	TO	None	168
O’Malley et al. [16]	2010	52	F	25	NR	NR	NA	PA	TORS	None	37
O’Malley et al. [16]	2010	75	F	60	NR	NR	NA	PA	TORS	Pharyngeal dehiscence	35
O’Malley et al. [16]	2010	58	F	43	NR	NR	NA	PA	TORS	None	33
O’Malley et al. [16]	2010	69	F	58	NR	NR	NA	PA	TORS	None	32
O’Malley et al. [16]	2010	35	F	31	NR	NR	NA	PA	TORS	Pharyngeal dehiscence	32
O’Malley et al. [16]	2010	64	F	32	NR	NR	NA	PA	TORS	None	31
O’Malley et al. [16]	2010	40	M	70	NR	NR	NA	PA	TO	None	16
Betka et al. [17]	2010	68	F	50	30	20	15.7	rPA	TO	None	NR
Betka et al. [17]	2010	37	M	70	50	40	73.3	rPA	TO	None	NR
Betka et al. [17]	2010	54	F	50	45	30	35.3	PA	TO	None	NR
Betka et al. [17]	2010	72	F	60	50	40	62.8	rPA	TO	None	NR
Betka et al. [17]	2010	36	F	50	40	40	41.9	PA	TO	None	NR
Betka et al. [17]	2010	52	F	55	50	35	50.4	CXPA	TO	None	NR
Betka et al. [17]	2010	53	F	55	30	30	25.9	PA	TO	None	NR
Kovačić et al. [18]	2012	45	F	50	50	NR	43.6	PA	TO	None	24
De Virgilio et al. [19]	2012	49	F	40	32	NR	17.9	PA	TORS	None	NR
De Virgilio et al. [19]	2012	43	M	55	40	NR	42.2	PA	TORS	None	NR
De Virgilio et al. [19]	2012	25	M	85	55	NR	138.7	PA	TP + TORS	None	NR
De Virgilio et al. [19]	2012	31	M	86	55	NR	142.0	PA	TP + TORS	None	NR
De Virgilio et al. [19]	2012	36	M	60	30	NR	37.7	PA	TP + TORS	None	NR
De Virgilio et al. [19]	2012	39	M	30	30	NR	9.4	PA	TORS	None	NR
De Virgilio et al. [19]	2012	57	F	40	30	NR	16.8	PA	TORS	None	NR
De Virgilio et al. [19]	2012	24	M	50	40	NR	34.9	PA	TP + TORS	None	NR
Hwang et al. [20]	2013	34	M	84	65	39	111.5	PA	TC + TO	Deep neck space seroma that was surgically drained and treated with intravenous antibiotics	3
Park et al. [21]	2013	42	M	NR	NR	NR	NA	PA	TORS	None	NR
Park et al. [21]	2013	31	M	NR	NR	NR	NA	PA	TORS	None	NR
Park et al. [21]	2013	39	M	NR	NR	NR	NA	PA	TORS	None	NR
Park et al. [21]	2013	47	F	NR	NR	NR	NA	PA	TORS	None	NR
Park et al. [21]	2013	57	F	NR	NR	NR	NA	PA	TORS	None	NR
Park et al. [21]	2013	29	M	NR	NR	NR	NA	PA	TORS	None	NR
Park et al. [21]	2013	21	F	NR	NR	NR	NA	PA	TORS	None	NR
Park et al. [21]	2013	54	F	NR	NR	NR	NA	PA	TORS	None	NR
Park et al. [21]	2013	30	M	NR	NR	NR	NA	PA	TORS	None	NR
Hussain et al. [22]	2014	49	F	60	50	35	55.0	PA	TO	None	72
Hussain et al. [22]	2014	60	F	50	40	28	29.3	PA	TO	None	42
Chen et al. [23]	2014	22	F	70	70	NR	119.7	PA	EATO	None	21
Chen et al. [23]	2014	33	F	70	60	NR	102.6	PA	EATO	None	16
Chen et al. [23]	2014	42	M	60	60	NR	75.4	PA	EATO	None	16
Chen et al. [23]	2014	53	M	40	40	NR	22.3	WT	EATO	None	9
Chen et al. [23]	2014	61	M	60	50	NR	62.8	PA	EATO	None	8
Samoy et al. [24]	2015	66	M	50	NR	NR	NR	PA	TORS	None	9
Li et al. [25]	2015	59	F	65	52	49	86.7	PA	EATO	None	NR
Li et al. [25]	2015	48	F	40	35	31	22.7	PA	EATO	None	NR
Li et al. [25]	2015	42	F	52	49	43	57.4	PA	EATO	None	NR
Iseri et al. [26]	2015	57	F	42	32	26	18.3	PA	EATO	None	6
Iseri et al. [26]	2015	53	F	27	31	37	16.2	NR	EATO	None	6
Iseri et al. [26]	2015	50	M	59	44	37	50.3	PA	EATO	Hyperemia of the skin of angulus mandibulae	6
Chan et al. [27]	2015	34	F	47	NR	NR	NA	PA	TORS	None	1
Chan et al. [27]	2015	51	F	60	NR	NR	NA	BCA	TORS	None	13
Chan et al. [27]	2015	43	F	54	NR	NR	NA	PA	TORS	None	15
Woo et al. [28]	2016	55	M	40	40	NR	22.3	PA	EATO	None	6
Dallan et al. [29]	2016	34	F	44	NR	NR	NA	PA	EATO	None	14
Dallan et al. [29]	2016	57	F	45	NR	NR	NA	PA	EATO	None	14
Dallan et al. [29]	2016	39	M	43	NR	NR	NA	PA	EATO	None	14
Casale et al. [30]	2016	67	M	56	43	22	27.7	WT	TO	Small collection of saliva, treated with drainage and intravenous antibiotics, solved after 5 days	NR
Boyce et al. [31]	2016	74	M	35	25	17	7.8	rPA	TORS	Yes	18
Boyce et al. [31]	2016	32	F	25	19	8	2.0	PA	TORS	None	13
Boyce et al. [31]	2016	70	M	39	23	15	7.0	MEC	TORS	None	50
Boyce et al. [31]	2016	54	M	40	12	10	2.5	PA	TORS	None	5
Boyce et al. [31]	2016	58	M	30	20	10	3.1	PA	TORS	None	20
Boyce et al. [31]	2016	73	F	40	35	7	5.1	BCA	TORS	None	2
Boyce et al. [31]	2016	48	M	70	70	30	77.0	PA	TC + TORS	None	11
Boyce et al. [31]	2016	60	F	42	37	33	26.9	PA	TORS	None	3
Boyce et al. [31]	2016	70	M	50	40	40	41.9	PA	TORS	None	1
Boyce et al. [31]	2016	78	F	22	15	6	1.0	O	TORS	None	3
Boyce et al. [31]	2016	65	M	28	12	10	1.8	PA	TORS	None	10
Boyce et al. [31]	2016	21	M	34	17	10	3.0	PA	TC + TORS	Yes	1
Boyce et al. [31]	2016	54	F	23	19	7	1.6	PA	TORS	None	10
Boyce et al. [31]	2016	72	F	50	42	30	33.0	PA	TORS	None	1
Wu et al. [32]	2018	52	F	33	NR	NR	NR	BCA	EATO	None	11
Wu et al. [32]	2018	48	F	60	40	30	37.7	BCA	EATO	None	20
Wu et al. [32]	2018	41	F	40	30	NR	16.8	BCA	EATO	None	37
Meng et al. [33]	2018	65	F	50	42	30	33.0	PA	TO	None	20
Meng et al. [33]	2018	67	M	50	40	30	31.4	CXPA	TO	None	14
Meng et al. [33]	2018	51	M	60	50	50	78.5	PA	TO	None	14
Meng et al. [33]	2018	45	F	43	40	35	31.5	PA	TO	None	14
Meng et al. [33]	2018	66	M	25	20	17	4.5	PA	TO	None	10
Meng et al. [33]	2018	51	F	50	45	30	35.3	PA	TO	None	8
Meng et al. [33]	2018	44	M	42	32	30	21.1	PA	TO	None	4
Meng et al. [33]	2018	28	M	58	42	32	40.8	PA	TO	None	3
Maglione et al. [34]	2018	23	M	72	62	38	88.8	PA	TORS	None	36
Maglione et al. [34]	2018	23	F	60	50	18	28.3	PA	TORS	None	39
Maglione et al. [34]	2018	44	M	60	30	25	23.6	PA	TO	None	42
Duek et al. [35]	2018	66	F	NR	NR	NR	111.0	PA	TORS	None	4
Duek et al. [35]	2018	78	F	NR	NR	NR	28.0	PA	TC + TORS	None	4
Duek et al. [35]	2018	42	M	NR	NR	NR	36.0	CXPA	TC + TORS	None	4
Duek et al. [35]	2018	69	F	NR	NR	NR	32.0	CXPA	TC + TORS	None	4
Duek et al. [35]	2018	30	M	NR	NR	NR	80.2	AC	TC + TORS	Marginal mandibular branch weakness, completely solved within 3 months	4
Duek et al. [35]	2018	33	M	NR	NR	NR	60.0	PA	TC + TORS	None	4
Duek et al. [35]	2018	64	F	NR	NR	NR	67.5	PA	TORS	None	4
Mani et al. [36]	2019	43	F	10	10	NR	0.3	PA	TO	None	8
Chen et al. [37]	2019	32	M	50	30	NR	26.2	PA	EATO	None	50
Chen et al. [37]	2019	34	M	50	40	NR	34.9	PA	EATO	None	69
Chen et al. [37]	2019	26	M	55	45	25	32.4	PA	EATO	None	44
Chen et al. [37]	2019	28	M	70	50	35	64.1	PA	EATO	None	50
Chen et al. [37]	2019	61	M	60	45	30	42.4	PA	EATO	None	31
Chen et al. [37]	2019	56	M	50	40	20	20.9	PA	EATO	None	50
Chen et al. [37]	2019	38	F	80	60	NR	134.0	PA	EATO	None	25
Chen et al. [37]	2019	45	M	62	38	NR	51.0	PA	EATO	None	12
Chen et al. [37]	2019	43	M	60	45	NR	56.5	PA	EATO	None	9
Chen et al. [37]	2019	38	F	58	37	30	33.7	PA	EATO	None	8
Shen et al. [38]	2020	72	M	35	30	20	11.0	WT	TO	None	23
Shen et al. [38]	2020	61	M	60	45	30	42.4	CXPA	TO	None	51
Moffa et al. [39]	2020	23	F	30	NR	NR	NA	PA	TORS	None	12
Li et al. [40]	2020	61	F	32	29	23	11.2	BCA	EATO	None	52
Li et al. [40]	2020	66	M	28	25	20	7.3	BCA	EATO	None	32
Yavuz et al. [41]	2021	70	F	53	48	34	45.3	PA	TO	None	NR
Voora et al. [42]	2021	37	M	40	30	NR	16.8	PA	TO	None	NR
Tsunoda et al. [43]	2021	62	M	70	NR	NR	NA	PA	EATO	None	>120
Tsunoda et al. [43]	2021	37	M	70	NR	NR	NA	PA	EATO	None	>120
Tsunoda et al. [43]	2021	65	M	60	NR	NR	NA	BCA	EATO	None	>120
Jbali et al. [44]	2021	50	M	52	38	NR	35.9	PA	TP + TO	House–Brackmann grade III facial palsy that solves using corticosteroid	NR
Shin et al. [45]	2022	60	F	36	NR	NR	NA	PA	TO	None	62
Salzano et al. [46]	2022	71	M	35	32	30	17.6	PA	TORS	None	6
Salzano et al. [46]	2022	63	F	40	20	20	8.4	PA	TORS	None	6
Salzano et al. [46]	2022	64	F	45	40	25	23.6	PA	TORS	None	6
Salzano et al. [46]	2022	23	M	40	30	20	12.6	PA	TORS	None	6
Salzano et al. [46]	2022	43	M	30	28	25	11.0	PA	TORS	None	6
Salzano et al. [46]	2022	33	F	35	30	20	11.0	PA	TORS	None	6
Salzano et al. [46]	2022	59	F	45	40	30	28.3	PA	TORS	None	6
Salzano et al. [46]	2022	25	F	35	28	20	10.3	PA	TORS	None	6
Salzano et al. [46]	2022	55	F	35	20	20	7.3	PA	TORS	None	6
Salzano et al. [46]	2022	27	M	35	30	30	16.5	PA	TORS	None	6
Salzano et al. [46]	2022	74	F	43	40	20	18.0	PA	TORS	None	6
Salzano et al. [46]	2022	58	F	40	35	20	14.7	PA	TORS	None	6
Salzano et al. [46]	2022	35	F	40	40	20	16.8	PA	TORS	None	6
Salzano et al. [46]	2022	42	M	38	25	25	12.4	PA	TORS	None	6
Lenzi et al. [47]	2022	76	F	30	23	21	7.6	PA	EATO	None	6
Cadena–Piñeros et al. [48]	2022	59	M	28	17	4	1.0	CXPA	TORS	None	6

Abbreviations: AC, adenocarcinoma; BCA, basal cell adenoma; CXPA, carcinoma ex pleomorphic adenoma; EATO, endoscopic assisted transoral; F, female; M, male; MEC, mucoepidermoid carcinoma; NA, not applicable; NR, not reported; O, oncocytoma; PA, pleomorphic adenoma; rPA, recurrent pleomorphic adenoma; TC, transcervical; TO, transoral; TORS, transoral robotic surgery; TP, transparotid; WT, Warthin tumor.
